# Tailoring Escherichia coli Chemotactic Sensing towards Cadmium by Computational Redesign of Ribose-Binding Protein

**DOI:** 10.1128/msystems.01084-21

**Published:** 2022-01-11

**Authors:** Hengyi Li, Changsheng Zhang, Xi Chen, Hantian You, Luhua Lai

**Affiliations:** a BNLMS, College of Chemistry and Molecular Engineering, Peking University, Beijing, China; b Center for Quantitative Biology, Academy for Advanced Interdisciplinary Studies, Peking University, Beijing, China; c Peking-Tsinghua Center for Life Sciences, Peking University, Beijing, China; Northwestern University

**Keywords:** bacterial chemotaxis, cadmium detection, computational design, protein engineering, ribose-binding protein

## Abstract

Periplasmic binding proteins such as ribose-binding proteins (RBPs) are involved in the bacterial chemotaxis two-component system. RBP selectively identifies and interacts with ribose to induce a conformational change that leads to chemotaxis. Here, we report the development of an engineered Escherichia coli (E. coli) strain expressing a redesigned RBP that can effectively sense cadmium ions and regulate chemotactic movement of cells toward a cadmium ion gradient. RBP was computationally redesigned to bind cadmium ions and produce the conformational change required for chemoreceptor binding. The successful design, CdRBP1, binds to cadmium ions with a dissociation constant of 268 nM. When CdRBP1 was expressed in the periplasmic space of E. coli, the bacteria became live cadmium ion hunters with high selectivity over other divalent metal ions. This work presents an example of making cadmium ions, which are toxic for most organisms, as an attractant to regulate cells movement. Our approach also demonstrates that RBP can be precisely designed to develop metal-detecting living systems for potential applications in synthetic biology and environmental studies.

**IMPORTANCE** Cadmium pollution is one of the major environmental problems due to excessive release and accumulation. New technologies that can auto-detect cadmium ions with good biocompatibility are in urgent need. In this study, we engineered the bacterial chemotaxis system to positively sense cadmium ions by redesigning ribose-binding protein (RBP) to tightly bind cadmium ion and produce the right conformational change for receptor binding and signaling. Our engineered E. coli cells can auto-detect and chase cadmium ions with divalent metal ion selectivity. Many attempts have been carried out to redesign RBP at the ribose binding site with little success. Instead of the ribose binding site, we introduced the cadmium binding site in the opening of the ribose binding pocket by a specially developed computational algorithm. Our design strategy can be applied to engineer live bacteria with autonomous detection and remediation abilities for metal ions or other chemicals in the future.

## INTRODUCTION

Bacteria rely on two-component systems to perceive and transduce environmental stimuli (1), including the chemotaxis system. This two-component chemotaxis system can accurately control cell motility in response to changes in environmental chemo-effectors (1–3). Cells with the chemotaxis system can efficiently and rapidly approach chemically favorable environments, such as those with higher concentrations of sugars, amino acids, and peptides, and avoid unfavorable ones, such as those consisting of ions and acids (4, 5). The ability of bacteria to sense and respond to novel effectors in their surroundings and to direct their migration would be useful in bioremediation (6, 7), biomedical deliveries. (8) and biosensor developments (9, 10).

Periplasmic binding proteins (PBPs) are a versatile superfamily of proteins involved in mediating bacterial chemotaxis (11). PBPs have been shown to interact with a variety of carbohydrates, anions, dipeptides, and amino acids that act as either attractant or repellent molecules (12). However, metal ions functioning as attractants through PBP binding have yet to be reported. Although PBPs bind a broad array of ligands, their three-dimensional structural folds are highly conserved. PBPs consist of two domains connected by a hinge region, with the ligand-binding site located at the interface of the two domains (13). Ligand binding elicits a conformational change that results in the formation of a protein-binding surface for chemoreceptor recognition (14, 15). Recently, a ligand was reported binding to the PBP, periplasmic glucose (or galactose) binding protein, and preventing the domain closure of this PBP. As a result, it acted as an antagonist that inhibits E. coli glucose chemotaxis (16).

Both the diversity of PBPs and their relatively flexible protein structures are helpful for the diverse chemotaxis ligands. Therefore, engineering of PBPs may give bacteria the ability to sense and respond to new effectors. Several attempts to construct PBP mutants that bind novel ligands at the ligand-binding sites have been reported (17, 18). However, most of these designs resulted in unfavorable protein folds or undesired binding abilities. Designing PBPs capable of recognizing and directing cell migration toward novel ligands still remains challenge.

Cadmium ion (Cd^2+^), as one of the heavy metals, is a significant threat to the environment due to accidental release. Strategies for cadmium detection and remediation in the environment have been developed (19–24). Emerging methods based on microorganisms for *in situ* bioremediation and biodetection of cadmium offer an alternative means for rapid, on-site heavy metal detection and remediation (25, 26). However, the movement of microorganisms is often hindered by the toxic nature of Cd^2+^, which results in inadequate distribution of cells in pollutant regions (25). Because the microorganism cells cannot actively seek cadmium, leading cadmium targeting ratio is still very low. Therefore, constructing bacterial chemotaxis behavior that could serve as self-guided motors to actively seek cadmium would be a preferable way to address these issues.

**FIG 1:**
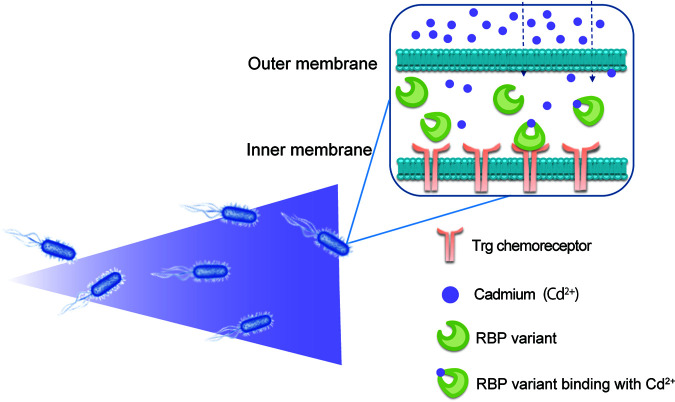
Schematic illustration of RBP variant-based E. coli chemotaxis mechanism. After the RBP variants expressed in the E. coli periplasmic region bind to cadmium ions, they undergo a conformational change from the open to the closed state that can interact with the Trg transmembrane receptor. This conformational change activates the signaling cascade that promotes E. coli chemotaxis toward cadmium ions.

In the present study, we generated an E. coli strain that can recognize and make a positive chemotaxis response to Cd^2+^, by engineering ribose-binding protein (RBP), which is one of the PBPs in E. coli. RBP selectively identifies and interacts with ribose (15, 27), which induces a change in protein conformation from an open to a closed state. The closed state of RBP allows further interaction with and activation of the Trg chemoreceptor (2, 28). Residues in RBP that are essential for chemotactic function (29), structural stability (30), periplasmic abundance, or signaling (31) have been identified. Herein, we developed a computational design algorithm systematically and efficiently searching for Cd^2+^-binding sites at the domain–domain interface in the RBP closed structure, in order to discover RBP mutants with the behavior of Cd^2+^-binding induced domain closure. With a series of experimental studies, we identified CdRBP1 as an RBP mutant that binds Cd^2+^ tightly and maintains the closed state conformation that is required for chemotaxis signaling. The engineered E. coli strain that expresses CdRBP1 in the periplasmic space exhibited positive chemotactic movement toward Cd^2+^ (Fig. 1).

## RESULTS

### The computational design strategy.

**FIG 2:**
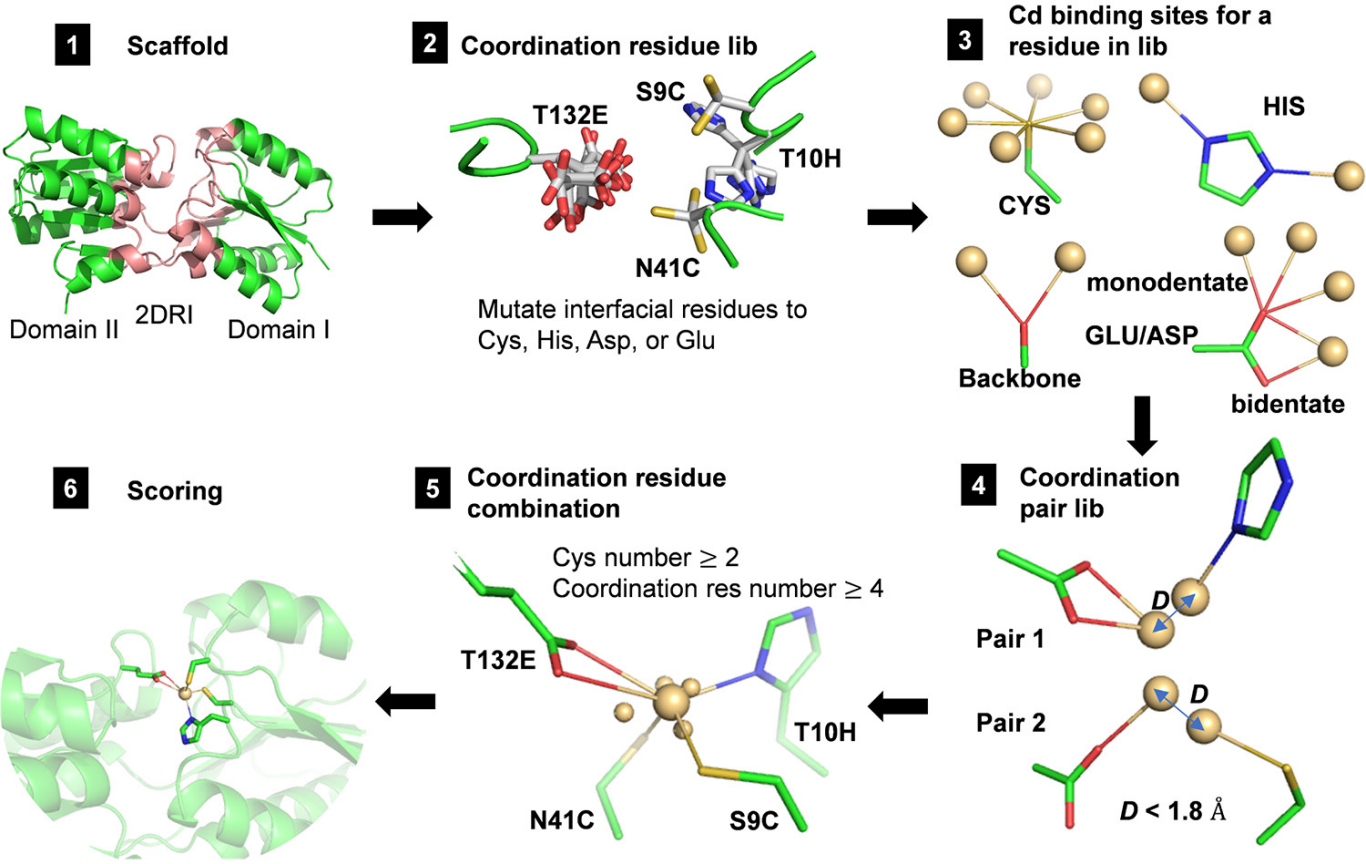
Computational process for designing Cd^2+^-binding ribose-binding protein (RBP) mutants. (1) The structure of the RBP in the holo state, 2DRI, was used as the starting scaffold protein. Only residues at the domain-domain interface (colored in pink) were mutable for Cd^2+^ coordination. (2) All of the mutable residues were mutated to Cd^2+^ coordination residues, including cysteine, histidine, aspartate, and glutamate, using a side chain assembly method. If the assembled side chain did not exhibit steric hindrance with surrounding residues, it was saved in the coordination residue library. The backbone oxygens were also saved to this library. (3) In this figure., T132E, S9C, T10H, and N41C are shown as examples from the coordination residue library. For each coordination atom in the residue library, Cd^2+^ binding position(s) were generated using a set of standard geometry parameters (Table S1, column 3). For the carboxyl group of aspartate or glutamate, one bidentate and three monodentate positions were generated. (4) If the distance (denoted as *D* in the figure) between two residues (at the standard coordination site) in the coordination library was less than 1.8 Å, and the two residues did not interfere with each other sterically, then this residue pair was saved to the coordination residue pair library. (5) A set-reduction algorithm was used to search for four- or five-residue combinations (containing at least two cysteines) that could coordinate on the same Cd^2+^ based on the coordination residue pair library. The average Cd^2+^ position (larger yellow sphere) was calculated from the individual coordination position for each residue (smaller yellow sphere). (6) The Cd^2+^ position was fine-tuned around the initial coordination, and a scoring function was used to rank the design results.

In a previous study, we have developed a computational algorithm, URANTEIN, to search for scaffold proteins for pockets that may accommodate uranyl ions and to subsequently design the uranyl binding site in the selected scaffold protein (32). For the selected scaffold protein, a library collecting the positions and conformations of all possible coordination residues was built first, and then a depth-first set-reduction algorithm (32, 33) was used for searching the combinations of these residues for the strong binding with uranyl ions. Using a similar strategy, in the present study we developed a computational algorithm for designing Cd^2+^ binding sites on a given scaffold protein. The corresponding program, CADEIN, can be accessed at: https://github.com/victorPKU/CADEIN. The computational process is illustrated in Fig. 2. The following is a general description of the method, and more details are given in the Materials and Methods section. Sulfur atoms in the cysteine side chain, carboxylate oxygen atoms in glutamate and aspartate, nitrogen atoms in the histidine side chain, and backbone carbonyl oxygen atoms are used as potential Cd^2+^ coordinating atoms according to the statistical analysis of the Cd^2+^-binding proteins in the Protein Data Bank (34).

In the present study, the closed structure of RBP (Protein Data Bank code 2DRI) was used as the protein scaffold (35), and the Cd^2+^ binding site was designed at the interface between the two domains. Each interfacial residue was computationally mutated to one of the Cd^2+^ coordination residues, cysteine, glutamate, aspartate, and histidine, using the side chain assembly method and the corresponding backbone-dependent rotamer libraries (36). All of these possible coordination residues at candidate sites formed the coordination residue library. Then, the positions for Cd^2+^ binding of each residue in the library were set. Coordination residues with Cd^2+^-binding positions close to each other (within 1.6 Å) were combined. All of the coordination pairs were identified and formed a coordination pair library. Based on the connectivity information of coordination residues in the pair library, a set-reduction algorithm (32, 33), which used a depth-first search procedure for the successive elimination of candidate combinations of coordination residues, was used to get the multi-residue-coordination solutions. To obtain high binding affinity mutants, we limited the search to solutions with four or five coordination residues containing at least two cysteines. Five high resolution crystal structures of Cd^2+^-binding proteins (resolution ≤1.5 Å, PDB codes: 5AI3 (37), 1R0I (38), 4CVS (39), 2OA9 (40), and 1WB4 (41)) were used as references for analyzing the Cd^2+^ coordination geometry and designing the scoring function. The average values of coordination bonds, bond angles, and dihedral angles (shown in Figure S1a in the supplemental material) in these structures were used as standard parameters and are presented in Table S1. If all these cadmium coordination geometry parameters of a solution meet the “acceptable” thresholds (Table S1), then the solution was retained. If all the parameters of a coordination residue in this solution meet the “perfect” thresholds, then the residue was assigned a higher score, otherwise it was assigned a lower score (Table S1). The summation of scores of all the coordination residues was used for fine-tuning the Cd^2+^-binding position and sorting all the retained solutions.

10.1128/msystems.01084-21.2 FIG S1 Illustration of designed cadmium binding sites on RBP. (a) Cadmium–residue coordination geometry parameters for cysteine, glutamate/aspartate bi- or mono-dentate, histidine, and backbone oxygen. (b) Illustration of designed cadmium binding sites on RBP domain-domain interfacial. Green cartoon: RBP scaffold. Sticks and spheres: coordination residues and cadmium binding sites. Coordination residues for each variant are labeled accordingly. (c) Introduction of A137Y and R141L into the computational search results. Ribose, orange color. R141L, green. (Arg), purple (Leu). A137Y, purple (Tyr). Hydrogen bonding interaction, black broken lines. Download FIG S1, TIF file, 2.1 MB. Copyright © 2022 Li et al. 2022 Li et al. https://creativecommons.org/licenses/by/4.0/ This content is distributed under the terms of the Creative Commons Attribution 4.0 International license.

10.1128/msystems.01084-21.9 TABLE S1 Cadmium coordination geometry in high resolution PDB structures (columns 4 and 5) and the parameters used for average coordination site generation (column 6) and scoring (columns 7–10). Download Table S1, DOCX file, 0.1 MB. Copyright © 2022 Li et al. 2022 Li et al. https://creativecommons.org/licenses/by/4.0/ This content is distributed under the terms of the Creative Commons Attribution 4.0 International license.

Using this algorithm, four Cd^2+^ coordination sites with corresponding mutations were identified, which were labeled as CdRBP1 to CdRBP4 (Fig. S1b). All the four Cd^2+^-binding sites were located around the ribose-binding cavity aperture and did not block ribose binding (Fig. S1b). In addition to these mutants, two more mutants were designed with manual inspections in which ribose binding was blocked through introduction of A137Y and R141L into the top two ranked computational search results, CdRBP1 and CdRBP2, respectively. The hydrogen bonding interactions between Arg 141 and ribose were removed by changing to Leucine, and the mutation A137Y result in bump with ribose (Fig. S1c). These mutants were labeled as CdRBP1m and CdRBP2m. All six designs were examined experimentally.

### RBP mutation leads to increased Cd^2+^ tolerance.

We assessed whether the RBP mutants could increase the toxicity tolerance of engineered E. coli strains on the plate by binding Cd^2+^ and reduce its harmful effects on E. coli cells. To this end, the RBP mutants were overexpressed into E. coli, and then cadmium adsorption of E. coli cells expressing wild-type and mutant RBP was measured using a plate screening assay. The growth of E. coli expressing wild-type RBP was significantly suppressed when exposed to 150 μM Cd^2+^ (Fig. S2a) due to the toxic effects of the ion. However, E. coli expressing CdRBP1, CdRBP3, CdRBP1m, and CdRBP2m remained viable under this condition (Fig. S2b), demonstrating that these mutants might be strongly associated with Cd^2+^. We chose these four mutants for further investigation.

10.1128/msystems.01084-21.3 FIG S2 The plate sensitivity assay for screening potential cadmium binding proteins. (a) Cadmium tolerance of E. coli cells expressing wild-type RBP at different cadmium concentrations. (b) Cadmium tolerance of E. coli cells expressing designed RBP variants at 150 μM Cd^2+^ concentration. Cells expressing CdRBP2, CdRBP4 showed no significant improvement of growth state compared with cells expressing wild-type RBP. Colony-forming units per milliliter (CFU mL^−1^) of the cultures were used for the calculation of the density of bacteria. Download FIG S2, TIF file, 1.8 MB. Copyright © 2022 Li et al. 2022 Li et al. https://creativecommons.org/licenses/by/4.0/ This content is distributed under the terms of the Creative Commons Attribution 4.0 International license.

### Cd^2+^ binding affinity of CdRBPs.

**FIG 3:**
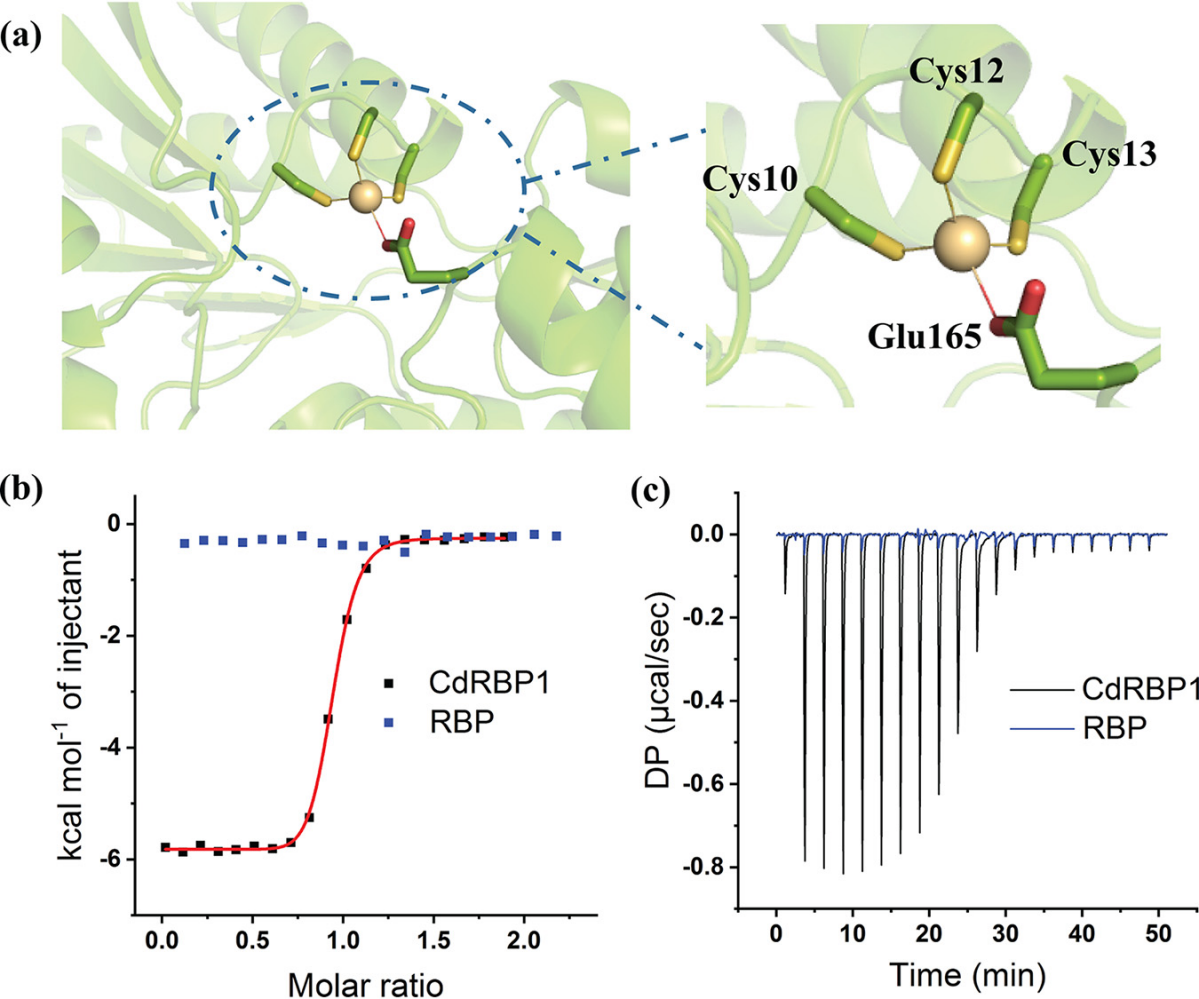
CdRBP1 binding to Cd^2+^. (a) Schematic of the designed Cd^2+^ binding site of CdRBP1 based on the RBP scaffold (left) and residues involved in Cd^2+^ coordination (right). (b) ITC measurements of CdRBP1 (black dots) and wild-type RBP (blue dots) binding with Cd^2+^. The CdRBP1 binding constant was determined by fitting the thermal data from one Cd^2+^ site binding model using MicroCal Origin software. (c) The heat released from each injection during titration of CdRBP1 (black line) and wild-type RBP (blue line).

Next, we purified and characterized the four CdRBP proteins that showed greatest potential for Cd^2+^ binding. The circular dichroism spectra of these four proteins showed secondary structure features similar to wild-type RBP (Fig. S5). We then performed isothermal titration calorimetry (ITC) experiments to measure the binding affinities and stoichiometric ratios of these proteins with Cd^2+^. The four mutants showed different Cd^2+^ binding behaviors (Fig. 3c and Fig. S3). The ITC titration data demonstrated that CdRBP1, CdRBP1m, and CdRBP2m bound Cd^2+^ in a 1:1 ratio while CdRBP3 exhibited two-site binding behavior (Table 1). The strongest binding protein, CdRBP1, bound Cd^2+^ with a dissociation constant of 268 nM. Under the same experimental conditions, wild-type RBP did not produce any binding signal in the ITC assay (Fig. 3b and 3c). We further verified Cd^2+^ binding of CdRBP1 and CdRBP2m by performing Fourier transform-mass spectrometry analysis. After incubation with Cd^2+^, both proteins showed a 110.2 Dalton increase in molecular weight, indicating complete incorporation of Cd^2+^ into the proteins (Fig. S4a-d). No increase of a Cd^2+^ molecular weight adduct was observed for the purified wild type RBP (Fig. S4e-f).

10.1128/msystems.01084-21.4 FIG S3 Secondary structure determination and *in vitro* binding characterization of cadmium binding proteins. ITC characterization of CdRBP3 (a), CdRBP1m (b), CdRBP2m (c) binding with Cd^2+^. (d) Circular dichroism (CD) spectra of RBP variants and wild-type RBP. Download FIG S3, TIF file, 2.7 MB. Copyright © 2022 Li et al. 2022 Li et al. https://creativecommons.org/licenses/by/4.0/ This content is distributed under the terms of the Creative Commons Attribution 4.0 International license.

10.1128/msystems.01084-21.5 FIG S4 FT-ESI mass spectrum analysis of Cd^2+^-free and Cd^2+^-binding proteins. Cd^2+^-free CdRBP1(a) and Cd^2+^-binding CdRBP1(b). Cd^2+^-free CdRBP2m (c) and Cd^2+^-binding CdRBP2m (d). Wild-type RBP (e) and wild-type RBP incubated with Cd^2+^ (f). Download FIG S4, TIF file, 1.6 MB. Copyright © 2022 Li et al. 2022 Li et al. https://creativecommons.org/licenses/by/4.0/ This content is distributed under the terms of the Creative Commons Attribution 4.0 International license.

**TABLE 1:** Characterization of the Cd^2+^-binding properties of RBP mutants by isothermal titration calorimetry

Variants	Mutant residues	Binding mode	Dissociation constant *K*_D_(M)	Stoichiometric ratio N^*a*^
CdRBP 1	D165E N13C N12C T10C	One site	(2.68 ± 0.25) × 10^−7^	1.00 ± 0.00
CdRBP 3	D165E N41H N13C N12C	Two sites	1 × 10^−5^, 1.1 × 10^−4^	ND^*b*^
CdRBP 1m	D165E N13C N12C T10C A137Y R141L	One site	(1.00 ± 0.33) × 10^−6^	1.00 ± 0.01
CdRBP 2m	I132E N41C T19H S9C A137Y R141L	One site	(2.19 ± 0.14) × 10^−6^	0.99 ± 0.02

a N is the apparent stoichiometry from experimental data fitting. ±, statistical error from fitting.

b ND, not determined. The stoichiometric ratio for CdRBP3 with two binding sites could not be fitted.

### Verification of the Cd^2+^ binding sites using alanine scanning mutagenesis.

In our computational models, CdRBP1 and CdRBP2m bound to Cd^2+^ through three coordination residues on the N-terminal domain and one glutamate residue on the C-terminal domain (Table 1). To verify the involvement of these residues, we individually replaced them with alanine and then measured the Cd^2+^ binding affinity. The ITC titration experiments indicated that the alanine substitution of Cys10 and Cys13 in CdRBP1 resulted in an approximately 20-fold decrease in binding affinity (Fig. S6). Alanine substitution of Glu165 resulted in a 5-fold decrease. Taken together, these results suggest that Cys10 and Cys13 contribute more to the association energy with Cd^2+^ than Glu165. Alanine substitution of the coordination residue Cys12 resulted in a change in stoichiometric ratio for Cd^2+^ binding, indicating that two types of cadmium binding sites with different binding affinities may present (Fig. S6). We propose that this residue may help orient the flexible loop (residues 8–13) into the proper conformation and promote single-site Cd^2+^ binding. These alanine scanning results suggested that CdRBP1 adopts the computationally designed coordination structure. However, for CdRBP2m, substitution of Glu132 had little effect on Cd^2+^-binding affinity, indicating that the three coordination residues from the N-terminal domain are the primary contributors to Cd^2+^ binding ability (Fig. S6).

### Chemotactic response of engineered E. coli toward cadmium ions.

**FIG 4:**
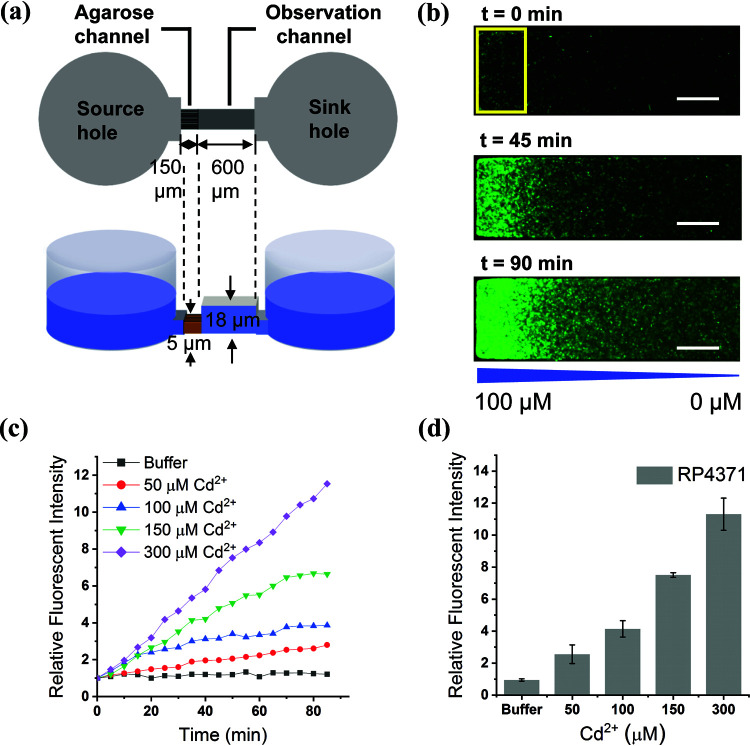
Chemotaxis of a CdRBP1-expressing E. coli strain toward Cd^2+^. (a) Schematic of the microfluidic device used to examine chemotaxis (side view and top view). (b) Responses of E. coli RP4371 to a Cd^2+^ gradient, recorded using fluorescence microscopy imaging at different times (scale bar, 100 μm). The Cd^2+^ gradient was from 0 μM to 100 μM, run through the observation channel. (c) Responses of the RP4371 strain to different Cd^2+^ concentrations as a function of time. The response is represented by relative fluorescent intensity, compared with *t* = 0 min at the same Cd^2+^ concentration in the analysis region (yellow rectangle, scale, 100 μm × 200 μm). The indicated concentrations are the source concentrations added to the source holes. (d) Final relative fluorescent intensities in the presence of different Cd^2+^ concentrations (mean ± SEM; N = 3).

Since CdRBP1 and CdRBP2m exhibited high Cd^2+^ binding affinity, we tested whether the E. coli cells with either of these two mutants expressed show chemotactic response toward Cd^2+^. The responses of the E. coli cells were measured using a designed microfluidic chip (Fig. 4a). In these experiments, green fluorescent protein (GFP)-labeled cells were added to a sink hole, and different concentrations of Cd^2+^ were introduced into the source holes (Fig. 4a). Our data demonstrate that the E. coli RP437 chemotaxis strain with periplasmic expression of CdRBP1 (RP4371) exhibited higher fluorescence intensity at the end of the observation channel compared with the blank buffer (Fig. 4b), and the fluorescence intensity increase was time dependent (Fig. 4c). These results indicated that RP4371 sensed the Cd^2+^ gradient and responded by accumulating gradually in the high-concentration region. The chemotactic response was also concentration dependent as the fluorescence intensity increased when the Cd^2+^ concentration in the source holes increased from 50 to 300 μM (Fig. 4d). The wild-type RBP-expressing RP437 strain did not respond to any Cd^2+^ concentration along the gradient (Fig. S7a), thus providing support for the involvement of CdRBP1 in the chemoattractant response toward Cd^2+^. No obvious chemotactic behavior was observed in cells expressing CdRBP2m under the same conditions (Fig. S7b-c).

We further assessed whether these two RBP mutants were exported to the periplasmic region, as this is mandatory for successful chemotactic sensing. We found that the expression level of CdRBP1 was higher than that of CdRBP2m in the periplasmic region of the engineered E. coli (Fig. S7d). The reduced CdRBP2m translocation was probably due to the N41C mutation, which was reported to disrupt the periplasmic translocation signal (31).

No chemotactic behaviors were observed for the CdRBP1-expressing E. coli VS2811 strain, which lacks the Trg receptor (Δ*trg*) (Fig. S7e), suggesting that the Trg chemoreceptor is required for the Cd^2+^ response. We also measured the effects of single coordination site mutations of CdRBP1 on E. coli chemotactic behavior. We found that all mutants had significantly reduced responses to Cd^2+^ (Fig. S7f-i).

10.1128/msystems.01084-21.8 FIG S7 Chemotactic response of engineered E. coli strains to cadmium gradient and investigation of proteins domain motion *in vitro*. Chemotactic response of cells expressing wild-type RBP (a) and expressing CdRBP2m (b) to different cadmium concentrations. The fluorescent intensities in the analysis region relative to the intensity at *t* = 0 min were plotted as a function of time for different Cd^2+^ gradients. (c) Fluorescent microscopic images of cells expressing CdRBP2m respond to buffer and 300 μM Cd^2+^, respectively (scale bar, 100 μm). (d) SDS-PAGE analysis of periplasmic protein level of E. coli RP437 strain expressing wild-type RBP, CdRBP1, and CdRBP2m compared with cells not induced with salicylate sodium. Black arrow indicates the expected position of these proteins detected in the periplasmic region. Chemotactic response of the E. coli VS281 strain expressing CdRBP1 (e), E. coli RPP437 expressing single site mutation of CdRBP1 to different cadmium concentrations (f-i). (j) ITC measurement of CdRBP1 binding with Zn^2+^ (red dots), Ni^2+^ (green dots), and Mg^2+^ (blue dots), respectively. The lines of different colors corresponding to the fitting results. (k) Relative changes in FRET ratio of purified wild-type RBP and CdRBP2m in the presence of ribose or Cd^2+^ compared with proteins in buffer alone (mean ± SEM; N = 5; ****, *P* < 0.0001; **, 0.001< *P* < 0.01, unpaired two tailed *t* test; ns, not significant). (l) The heat released from each injection during titration of CdRBP1 (black line) with Zn^2+^. (m) Relative changes in FRET ratio of purified wild-type RBP and CdRBP1 in the presence of ribose or Zn^2+^ compared with proteins in buffer alone (mean ± SEM; N = 5; ****, *P* < 0.0001, unpaired two-tailed *t* test; ns, not significant). Download FIG S7, TIF file, 1.2 MB. Copyright © 2022 Li et al. 2022 Li et al. https://creativecommons.org/licenses/by/4.0/ This content is distributed under the terms of the Creative Commons Attribution 4.0 International license.

### Measurement of domain closure using fluorescence resonance energy transfer assay.

**FIG 5:**
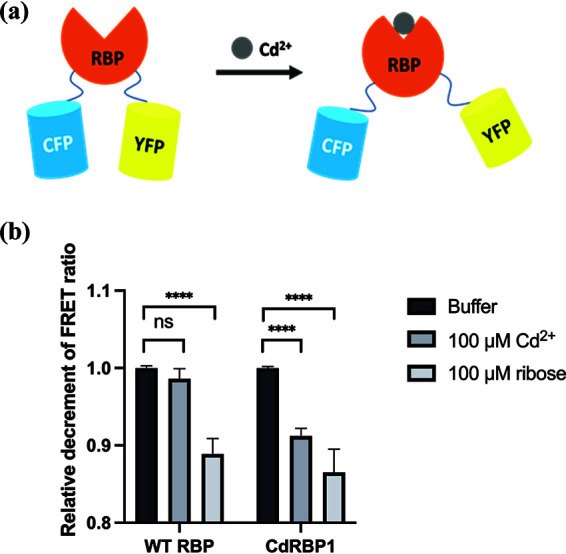
Interdomain conformational changes were measured using a FRET assay. (a) Schematic illustration of the FRET assay based on RBP structure. (b) Changes in FRET ratio of purified wild-type RBP and CdRBP1 in the presence of ribose or Cd^2+^ compared with proteins in buffer alone (mean ± SEM; N = 5; ****, *P* < 0.0001, unpaired two-tailed *t* test; ns, not significant).

To investigate the Cd^2+^-induced conformational changes in CdRBP1, we fused a cyan fluorescent protein (CFP) and a yellow fluorescent protein (YFP) to the N- and C-termini of the protein, respectively. Due to the relative positions of the two domains of RBP, fluorescence resonance energy transfer (FRET) occurs when the RBP structure is open (the apo state without ligand binding) (42, 43). Upon Cd^2+^ binding, domain closure occurs, which drives the two fluorescent proteins away from each other, resulting in a decreased FRET signal (Fig. 5a),

As shown in Fig. 5b, CdRBP1 exhibited a lower FRET ratio upon Cd^2+^ binding that was similar to RBP upon ribose binding, implying that CdRBP1 and the wild-type protein undergo similar conformational changes.

### Metal ion selectivity of the engineered RP4371 strain.

**FIG 6:**
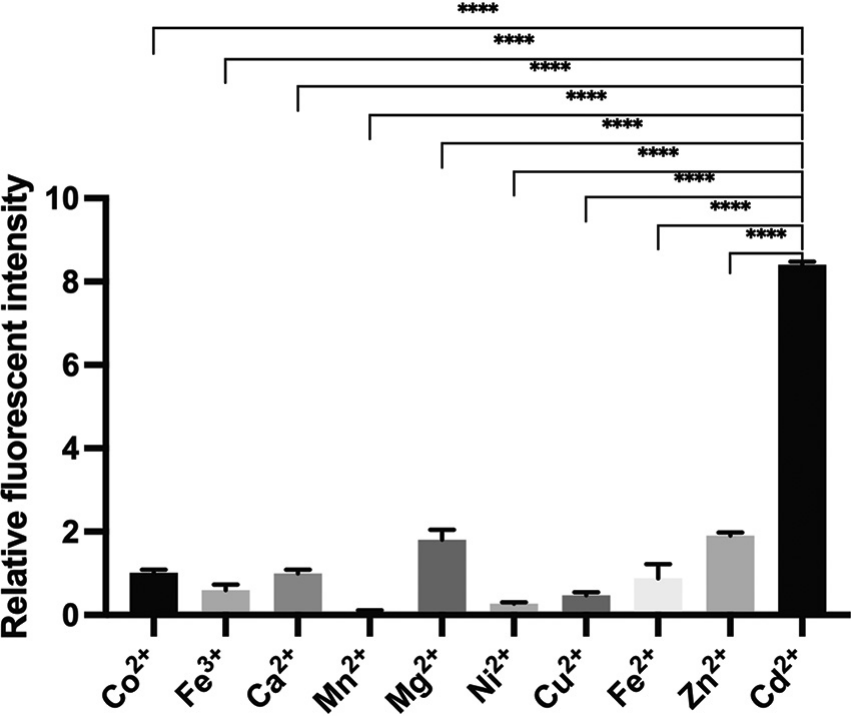
CdRBP1 binding is selective for Cd^2+^. A microfluidic chemotaxis assay was performed to examine the response of E. coli RP4371 to 250 μM of the indicated metal ion (mean ± SEM; N = 3; ****, *P* < 0.0001, unpaired two-tailed *t* test).

We further tested the metal ion selectivity of our engineered Cd^2+^-sensing RP4371 strain. Among the 10 commonly encountered metal ions, including Co^2+^, Fe^3+^, Ca^2+^, Mn^2+^, Mg^2+^, Ni^2+^, Cu^2+^, Fe^2+^, and Zn^2+^, RP4371 only significantly responds to Cd^2+^ in the microfluidic chemotaxis assay (Fig. 6). The great selectivity for Cd^2+^ of the engineered E. coli strain might result from the binding specificity of CdRBP1 to Cd^2+^ and the correct conformational changes of CdRBP1 caused by the Cd^2+^ binding. We used ITC to measure the *in vitro* binding affinity of CdRBP1 to metal ions with similar coordination properties to Cd^2+^. Compared to its affinity to Cd^2+^, CdRBP1 showed a much weaker binding affinity to Ni^2+^ (approximately 10-fold lower) and no binding to Mg^2+^ (Fig. S7j). For the most similar ion, Zn^2+^, our ITC study showed that it binds CdRBP1 with exothermic profile followed by endothermic profile with increasing zinc ion concentration (Fig. S7l), indicating that there are two types of zinc binding sites in CdRBP1. The first binding site, which is the stronger binding site, was fitted, and the dissociation constant was 5.68 × 10^−7^ M, which is about 2-fold weaker than that of Cd^2+^, as shown in Fig. S7j. FRET assay demonstrated that Zn^2+^ binding did not produce similar conformational change in CdRBP1 (Fig. S7m), which is consistent with the chemotaxis result.

## DISCUSSION

Bacterial chemotactic intelligence provides a natural system for reconstructing sensory robots that autonomously hunt for novel ligands. Here, we present a strategy for designing and developing Cd^2+^-binding proteins based on the E. coli RBP, a periplasmic binding protein responsible for chemotaxis. By employing a computational design method to search for Cd^2+^ binding sites that accommodate proper coordination geometry, we obtained four Cd^2+^-binding RBP variants. Using a FRET assay, we confirmed that in one of the computationally designed proteins, namely, CdRBP1, Cd^2+^ binding induced domain closure reproducing the conformational change of the wild-type RBP with ribose-binding. This combination of computational and experimental methods represents a successful example of incorporation of Cd^2+^ into the RBP scaffold.

Many attempts have been made to construct RBP mutants that bind to novel ligands at the ribose-binding site. However, due to the tight binding of ribose and the complicated conformational changes that occur after ribose binding, some mutations on RBP result in misfolded or poorly translocated proteins (31), making these designs produce undesired binding modes or binding abilities (17, 18). Recently, RBPs were engineered to bind compounds that are structurally similar to ribose at the ribose binding site, by mutant library screening strategy (44). The resulting RBP variants were shown to bind with ligand *in vitro*. Unfortunately, the binding strength is much weaker and the effect on chemotaxis signal transduction pathway is unclear. These previous efforts indicate that engineering RBP to bind new ligands within the ribose-binding site and further related to function remains challenging. Instead of modifying the ribose binding site, we designed a Cd^2+^-binding site in RBP at a new site, that is, the lids of the domain closure structure. Our mutation studies showed that the right coordination of Cd^2+^ at this site is important for binding and to induce the necessary domain closure conformational changes required by the chemotaxis function. For example, when we tried to improve Cd^2+^ binding affinity of CdRBP1 by mutating D165 to Cys and elongating the nearby loop to make the sulfur atom reaching Cd^2+^ (Fig. S5), the mutants tend to bind two Cd^2+^ ions (Fig. S5d). The mutation of 10C on CdRBP1 to 41H (resulting CdRBP3) also changed the binding stoichiometry to two Cd^2+^ ions with one protein (Table 1, Fig. S3). Alanine substitution of each coordination residue on CdRBP1 resulted in either decrease of binding affinity or binding mode changes (Fig. S6). These mutants showed significantly reduced chemotactic responses to Cd^2+^ in cells (Fig. S7). The CdRBP2m variant possesses favorable binding affinity with Cd^2+^ *in vitro* but showed no chemotactic sensing in cells (Fig. S7b). One possible reason is the disrupted periplasmic translocation ability. In addition, another plausible reason is due to lack of interaction with the Trg receptor. We checked the binding of CdRBP2m with ribose using FRET assay. As shown in Fig. S7k, CdRBP2m exhibited less decrement of FRET ratio upon ribose binding compared to wild-type binding with ribose, implying that mutations in CdRBP2m impair ribose binding-induced conformation change. When CdRBP2m binds Cd^2+^, an opposite change in the FRET ratio was observed, indicating a more open conformation that is distinct from the closed conformation required by Trg binding. Thus, Cd^2+^ binding of CdRBP2m does not produce necessary conformational change required for chemoreceptor binding; this should be the main reason for its lack of chemotaxis activity (31). Taken together, besides tight binding, proper conformational change and the right translocation to the periplasmic region of variants are mandatory for the successful chemotactic sensing.

Chemotaxis signaling produced by RBP requires its conformational change from open conformation to close conformation. Thus, only strong binding does not necessarily result in chemotaxis signaling. The large conformational change itself produces entropy loss during binding. In addition, the interface between the two domains of RBP are dominantly composed of polar residues, which are more solvent exposed on protein opening status (27). The change of protein conformation induced by cadmium would squeeze out water molecules. The penalty of desolvation of polar groups next to the protein-solvent interface upon binding would give rise to the decrease of observed binding affinity. Natural cadmium binding proteins, such as metallothionein, have rigid structures that do not change after cadmium binding. These proteins usually use multiple thiols from cysteine residues to bind Cd^2+^, forming Cd^2+^ thiolate clusters with high binding strength. Thus, both the large conformational change of RBP and the burying of polar groups upon cadmium binding produce entropic loss that result in weakened binding compared to natural cadmium binding proteins that use rigid binding sites.

10.1128/msystems.01084-21.6 FIG S5 CD spectra and ITC characterization of the optimized CdRBP1s. (a), (b), and (c) show the CD spectrum of CdRBP1-1, CdRBP1-2, and CdRBP1 (A102G), respectively. CdRBP1-2 exhibited a large structural change compared to the other two mutants. (d) Cadmium binding affinity of CdRBP1-1 showed two sequential binding sites. (e) Cadmium binding affinity of CdRBP1 (A102G). Download FIG S5, TIF file, 2.6 MB. Copyright © 2022 Li et al. 2022 Li et al. https://creativecommons.org/licenses/by/4.0/ This content is distributed under the terms of the Creative Commons Attribution 4.0 International license.

10.1128/msystems.01084-21.7 FIG S6 ITC characterization of the CdRBP1 alanine scanning mutagenesis in the cadmium binding site. (a) CdRBP1 (C10A). (b) CdRBP1 (C12A). K_1a_ and K_2a_ represent the association constants of different binding steps. (c) CdRBP1 (C13A), (d) CdRBP1 (E165A). ITC characterization of the CdRBP2m single alanine scanning mutagenesis in the cadmium binding site. (e) CdRBP2m (C9A), (f) CdRBP2m (H10A), (g)CdRBP2m (C41A), (h) CdRBP2m (E132A). Download FIG S6, TIF file, 2.6 MB. Copyright © 2022 Li et al. 2022 Li et al. https://creativecommons.org/licenses/by/4.0/ This content is distributed under the terms of the Creative Commons Attribution 4.0 International license.

Using our in-house-designed microfluidic device for the bacterial chemotaxis assay, we found that engineered E. coli expressing CdRBP1 exhibited chemotactic behavior along a Cd^2+^ gradient with divalent metal ion selectivity. Our approach provides a strategy for designing novel bacterial chemotaxis properties based on PBPs. Heavy metals are chemorepellents for most natural microbes; our work demonstrates that it is possible to engineer bacteria with chemoattractant properties toward metal ions (Cd^2+^). With the benefit of the bacteria moving toward Cd^2+^, our engineered E. coli systems may improve the current Cd^2+^ microbial sensors and remediation strategies, which are vulnerable due to the toxic nature of Cd^2+^ (25). In increased levels of contaminated environment, durable cadmium detection and remediation might be realized by incorporation of proteins with high cadmium-binding-affinity or cadmium detection circuits into our engineered E. coli cells.

## MATERIALS AND METHODS

### Computational design of Cd^2+^ binding proteins.

The structure of the RBP was divided into two domains, domain I (residues 1–103, 236–263) and domain II (residues 104–235, 264–271). Only residues on the domain–domain interface were mutable for cadmium coordination. Prolines and residues in the hinge region (102–104, 234–236, 263–265), which are important for maintaining proper conformation, were set to be nonmutable. If a Cα atom in a domain was within 12 Å of any Cα atom of the other domain, it was defined as interfacial. A backbone-dependent rotamer library from Dunbrack’s lab was used for side chain assembly (36). The T132E, S9C, T10H, and N41C mutations are shown in Fig. 2. Before side chain assembly, backbone conformations were slightly perturbed using the backrub method for considering backbone flexibility (45), and the backrub angle was set as −6°, 0°, or +6°.

The geometry parameters in Table S1 were used to calculate the standard coordination sites for a single residue. Residues with cadmium coordination sites close to each other (within 1.6 Å) were combined (as Fig. 2 step 5 illustrates) and screened with the “acceptable” thresholds for each geometry parameter (bond, angle, or dihedral) in Table S1. A set-reduction algorithm was used for the combinations search based on the coordination residue pair library. There is a library reduction process on the initial coordination residue pair library. Using the initial coordination pair library, the maximum number of possible co-coordination residues for a given residue in the coordination library was calculated. If the maximum co-coordination number of a residue was less than 3, it was removed from the library. After reducing the coordination residue library, the coordination residue pair library was updated.

Solutions meeting the “acceptable” thresholds were scored by adding up the coordination score of each residue. The coordination scoring function expresses the quality of residue conformation and coordination geometry as

(1) $$\mathrm{Score}=\underset{i}{\sum }Q\mathrm{ualit}y_{i}\;+\;\;\mathrm{CoordinationScor}e_{i}$$

The quality of residue conformation includes side chain conformation, backbone conformation, and coordination type terms as

(2) $$\mathrm{Qualit}y_{i}=0.9\times \log\left(\mathrm{pi}\right)\;-\;2\;\times \;\frac{\left|\theta_{1,3}\right|}{6.0}\;+\;\text{wi}$$

where *p* is the probability of the side chain rotamer and θ_1_,_3_ is the backbone backrub angle. The coordination type weight, w, was defined as Cys, 0.8; His, 1.0; Asp/Glu, 0.8; and backbone oxygen 3.0.

The coordination geometry was scored as a two-level function, namely, perfect and acceptable. There was a threshold for all types of coordination at these two levels (see Table S1). For perfect and acceptable coordination, a discrete score (see Table S1 for the different types of coordination) was added to the total score. The location of the cadmium site was fine-tuned around the initial coordination in a 0.8 × 0.8 × 0.8 Å lattice within a 0.1 Å space.

### Plasmid construction for RBP and variants.

The genes encoding the RBP and RBP variants were cloned into expression vectors by PCR followed by restriction enzyme digestion and ligation. All RBP variants were obtained using a site-directed mutagenesis kit (TIANGEN). All sequences were confirmed by DNA sequencing. See the SI for experimental details.

### Plate sensitivity assay.

The number of E. coli strains overexpressing wild type RBP and variants were normalized and subjected to six 10-fold serial dilutions. Samples (10 μL) of each were spotted onto LB agar plates containing different cadmium concentrations. All plates were incubated at 37°C for 16 h before measurement. See the supplemental material for experimental details.

### Protein expression and purification.

The recombinant plasmids expressing wild-type RBP, cadmium-binding proteins, or the single alanine substitution mutants in pET-28a were transformed individually into BL21(DE3) strains for protein expression. Cell lysate was applied to HisTrap columns (GE Healthcare Life Sciences) and the N-terminal 6× His-tag was removed by on-column digestion. The target protein was eluted and then applied to a Sephacryl S-200 HR column for further purification. Proteins with FRET pairs were expressed and purified using a similar method.

### Circular dichroism spectroscopy.

The far-UV CD spectra (190–260 nm) were recorded using MOS 450 AF/CD (Biologic, France) at room temperature. Protein samples were diluted to a final concentration of 0.1 mg/mL using 20 mM phosphate potassium buffer (pH 7.3) containing 150 mM KCl. Samples were scanned in a quartz cuvette with the pathlength of 1 mm at a spectral bandwidth of 1 nm. Signals from three scans were averaged.

### Isothermal titration calorimetry (ITC) assay.

An iTC200 Microcalorimeter (MicroCal, USA) was used to measure the binding affinity (Ka) of cadmium with purified wild-type RBP and the designed variants and single alanine mutants. All data were analyzed using MicroCal Origin software. See the supplemental material for details.

### FT-mass spectrometry analysis.

The total mass of cadmium-free proteins and cadmium-binding proteins was determined by using a Fourier Transform Ion Cyclotron Resonance mass spectrometer with electrospray ionization (SolariX XR, Bruker). Cadmium-binding proteins were prepared by incubating the proteins with CdCl_2_ for 30 min at 4°C before desalting. All proteins were desalted by ultrafiltration with ddH_2_O three times before measurement.

### FRET assay.

The FRET protein samples were diluted to 5 μM and then incubated with excessive cadmium ions or ribose to a final concentration of 100 μM at room temperature for 20 min. ECFP was excited at 433 nm, and then the emission fluorescence signal intensity was measured at 475 nm and 527 nm for ECFP and EYFP, respectively. Fluorescence signals were recorded on a microplate reader (Synergy, Bioteck). The FRET ratio was calculated by dividing the fluorescent emission intensity of ECFP by that of EYFP. Proteins diluted in blank buffer were measured as a control.

### Microfluidic experiments.

Details of the microfluidic device and operating principles are shown in Fig. 5a and the supplemental material. E. coli cells expressing GFP protein were loaded into the sink holes and then allowed to diffuse into the observation channel to reach a steady state. Cadmium and other divalent metal ions were added into the source holes. The responses of the cells to different concentrations of metal ions were observed by microscopy for 1 to 2 h. The response of the cells was quantified by measuring the fluorescent intensity of the analysis region using the NIH Image J software (46).

### Analysis of protein expression in periplasmic region by osmotic shock.

Recombinant plasmids expressing RBP and variants with N-terminal signal peptide sequence were transformed into E. coli RP437 and induced with sodium salicylate. The proteins expressed in the periplasmic region were extracted using an osmotic shock procedure (46, 47). The extracted protein samples were then analyzed by 10% SDS-polyacrylamide gel electrophoresis (PAGE). See the supplemental material for experimental details.

### Strains and plasmids.

Information regarding the genotypes, phenotypes, and sources of the bacterial strains and plasmids used in this study are listed in Table S2.

10.1128/msystems.01084-21.10 TABLE S2 Strains used in the microfluidic experiment study. Download Table S2, DOCX file, 0.01 MB. Copyright © 2022 Li et al. 2022 Li et al. https://creativecommons.org/licenses/by/4.0/ This content is distributed under the terms of the Creative Commons Attribution 4.0 International license.

### Data availability.

The source code of the design program and the designed structure models are available at https://github.com/victorPKU/CADEIN.

10.1128/msystems.01084-21.1 TEXT S1 Supplementary materials and methods. Download Text S1, DOCX file, 0.03 MB. Copyright © 2022 Li et al. 2022 Li et al. https://creativecommons.org/licenses/by/4.0/ This content is distributed under the terms of the Creative Commons Attribution 4.0 International license.
