# Supplementary material for: Tailoring Escherichia coli Chemotactic Sensing towards Cadmium by Computational Redesign of Ribose-Binding Protein
Source: mSystems. 2022 Jan 11;7(1):e01084-21. doi: 10.1128/msystems.01084-21 (PMC8751387; doi:10.1128/msystems.01084-21)
Supplement: TABLE S1 [file msystems.01084-21-st001.docx]

| **Table S1. Cadmium coordination geometry in high resolution PDB structures (columns 4 and 5) and the parameters used for average coordination site generation (column 6) and scoring (columns 7-10).** | | | | | | | |  |  |
| --- | --- | --- | --- | --- | --- | --- | --- | --- | --- |
| Coordination residue | Reference structure (PDB ID, chain, residue #)*** | Coordination geometry*†* | Average | Min—max | Standard coordination | Perfect threshold | Score for perfect | Acceptable threshold | Score for acceptable |
| Cysteine | 5AI3 A 5, 8, 38, 41; 1R0I A 6, 9, 39, 42 | S𝛾-Cd bond (b, Å) | 2.51 | 2.46—2.55 | 2.5 | 2.35-2.65 | 24 | 2.25-2.75 | 10 |
|  |  | C𝛽-S𝛾-Cd angle (𝜃, º) | 102.0 | 98.7—107.3 | 102.0 | 90-120 |  | 85-130 |  |
|  |  | C𝛼-C𝛽-S𝛾-Cd dihedral (𝜑, º) | —— | —— | 0, ±60, ±120, 180 | 0-180 |  | 0-180 |  |
| Histidine | 4CVS A 54, 130; 2OA9 A 59, B 59; 1WB4 A 1076,1081,1083 | N𝛿-Cd or N𝜀-Cd bond (b,Å)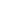 | 2.34 | 2.29—2.36 | 2.34 | 2.20-2.50 | 16 | 2.10-2.60 | 8 |
|  |  | Distance difference of C𝛾(or C𝛿) and C𝜀 to Cd (\|d_1_-d_2_\|, Å) | 0.16 | 0.07—0.29 | 0 | 0-0.25 |  | 0-0.40 |  |
|  |  | Angle between N-Cd bond and the imidazole ring (𝜑, º) | 12.1 | 3.7—30.2 | 0 | 0-30 |  | 0-45 |  |
| Aspartate or glutamate bidentate coordination | 4CVS A 18, 94; 2OA9 A 234, 238, B 234, 238; 1WB4 A 1079 | O𝛿-Cd or O𝜀-Cd bond (b_1_, b_2_, Å) | 2.37 | 2.27—2.46 | 2.35 | 2.20-2.50 | 20 | 2.10-2.60 | 10 |
|  |  | Distance difference of the two C𝛾(or C𝛿) to Cd (\|b_1_-b_2_\|, Å) | 0.1 | 0.03—0.16 | 0 | 0-0.25 |  | 0-0.40 |  |
|  |  | Angle between O-Cd bond and the carboxyl plane (𝜑, º) | 3.3 | 0.3—6.5 | 0 | 0-30 |  | 0-45 |  |
| Aspartate or glutamate monodentate coordination | 4CVS A 51, 127; 2OA9 A 203, B 203; 1WB4 A 894 | O-Cd or O-Cd bond (b, Å) | 2.31 | 2.24—2.36 | 2.35 | 2.20-2.50 | 12 | 2.10-2.60 | 6 |
|  |  | C-O-Cd angle (𝜃, º) | 131.7 | 121.5—139.7 | 130, 180 | 110-150 |  | 95-165 |  |
|  |  | Angle between O-Cd bond and the carboxyl plane (𝜑, º) | 32.7 | 2.5—68 | 0 | 0-50 |  | 0-70 |  |
| Backbone oxygen | 1WB4 A 926,929,985,1076 | O-Cd bond (b, Å) | 2.33 | 2.23—2.40 | 2.35 | 2.20-2.50 | 16 | 2.10-2.60 | 8 |
|  |  | C-O-Cd angle (𝜃, º) | 149.3 | 138.9—157.5 | 150 | 130-170 |  | 115-180 |  |
| * X-ray structure resolution (Å): 5AI3, 1.02; 1R0I, 1.5; 4CVS, 1.39; 2OA9, 1.5; 1WB4, 1.4. | | | | |  |  |  |  |  |
| † The coordination geometry parameters in the third column is shown in Fig S1. | | | | |  |  |  |  |  |
|  |  |  |  |  |  |  |  |  |  |
|  |  |  |  |  |  |  |  |  |  |
