# Supplementary material for: Tailoring Escherichia coli Chemotactic Sensing towards Cadmium by Computational Redesign of Ribose-Binding Protein
Source: mSystems. 2022 Jan 11;7(1):e01084-21. doi: 10.1128/msystems.01084-21 (PMC8751387; doi:10.1128/msystems.01084-21)
Supplement: TABLE S2 [file msystems.01084-21-st002.docx]

Table S2. Strains used in in the microfluidic experiment study

| Strains | Genotype or phenotype | Notes | References |
| --- | --- | --- | --- |
| RP437 | *thr-1leuB6 his-4 metF59 eda-50 rpsL136* | Wild type *E. coli* | J. S. Parkinson, S. E. Houts, *J Bacteriol* 1982, *151*, 106-113. |
| RP4371 | RP437/pSN77-CdRBP1,  Cam^r^, Amp^r^ | RP437 containing pSN77- CdRBP1 plasmid | this study |
| RP4372m | RP437/pSN77-CdRBP2m,  Cam^r^, Amp^r^ | RP437 containing pSN77- CdRBP2m plasmid | this study |
| RP437(wild type RBP) | RP437/pSN77 Cam^r^, Amp^r^ | RP437 containing pSN77 plasmid | this study |
| RP4371(10A) | RP437/pSN77-CdRBP1(10A),  Cam^r^, Amp^r^ | RP437 containing pSN77- CdRBP1(10A) plasmid | this study |
| RP4371(12A) | RP437/pSN77-CdRBP1(12A),  Cam^r^, Amp^r^ | RP437 containing pSN77- CdRBP1(12A) plasmid | this study |
| RP4371(13A) | RP437/pSN77-CdRBP1(13A),  Cam^r^, Amp^r^ | RP437 containing pSN77- CdRBP1(13A) plasmid | this study |
| RP4371(165A) | RP437/pSN77-CdRBP1(165A),  Cam^r^, Amp^r^ | RP437 containing pSN77- CdRBP1(165A) plasmid | this study |
| VS281 | RP437 *Δtrg* ::Tn10 | RP437 strain lacks chemoreceptor Trg | Gift from Prof. Victor Sourjik |
| VS2811 | VS281/pSN77-CdRBP1,  Cam^r^, Amp^r^ | VS281 with pSN77-CdRBP1 plasmid | this study |

*pSN77 has *rbsB gene* cloned, expressing *E. coli* wild type RBP. (S. Neumann, C. H. Hansen, N. S. Wingreen, V. Sourjik, *Embo J* 2010, *29*, 3484-3495.)
