# Supplementary material for: Tailoring Escherichia coli Chemotactic Sensing towards Cadmium by Computational Redesign of Ribose-Binding Protein
Source: mSystems. 2022 Jan 11;7(1):e01084-21. doi: 10.1128/msystems.01084-21 (PMC8751387; doi:10.1128/msystems.01084-21)
Supplement: TEXT S1 [file msystems.01084-21-t0001.docx]

**Supplementary Text**

Tailoring *Escherichia coli* Chemotactic Sensing towards Cadmium by Computational Redesign of Ribose-binding Protein

Hengyi Li^a^*^†^*, Changsheng Zhang^a^*^†^*, Xi Chen^b^, Hantian You^a^ and Luhua Lai^a,b,c,*^

^a^BNLMS, College of Chemistry and Molecular Engineering, Peking University, Beijing 100871 (P.R. China), ^b^Center for Quantitative Biology, Academy for Advanced Interdisciplinary Studies, Peking University, Beijing 100871 (P.R. China), ^c^Peking-Tsinghua Center for Life Sciences, Peking University, Beijing 100871(P.R. China)

*Correspondence should be addressed to Luhua Lai

**Email:**  [lhlai@pku.edu.cn](mailto:lhlai@pku.edu.cn)

*†* These authors contributed equally to this work

Supplementary Materials and Methods

**Plasmids construction for RBP and variants**

The gene encoding ribose binding protein obtained by PCR reaction from *E. coli* K-12 strain and was then cloned into pET-21a expression vector using *Nde* I and *Xho* I two restriction enzyme sites. The RBP sequence was confirmed by DNA sequencing. The designed RBP variants and alanine mutations were obtained by using site-directed mutagenesis kit (TIANGEN). CdRBP1 and CdRBP2m proteins and their single alanine substitution mutant genes were constructed into pET-28a vector via *Nde* I and *Xho* I with N terminal 6*His tag. The expression gene with signal peptide of CdRBP1 and CdRBP2m were constructed by two steps PCR. The gene encoding signal peptide and genes encoding CdRBP1 and CdRBP2m were both amplified. Then these two purified fragments were connected with signal peptide by circular polymerase extension cloning(*1*). The PCR products were digested by *Spe* I and *BamH* I two restriction enzymes, and later inserted into pSN77 vector(*2*), which was a gift from Professor Victor Sourjilk, for proteins expression into *E. coli* periplasmic region.

**Plate sensitivity assay of designed cadmium binding proteins**

The *E. coli* strains BL21 (DE3) containing wild type RBP and variants plasmids were grown at 37 °C overnight in LB medium with 50 µg mL^-1^ ampicillin and were inoculated into fresh LB medium with 1:100 dilution. Cultures were grown at 37 °C till OD _600_ =0.6-0.8 and induced with 0.5 mM IPTG. After further incubation at 28 °C for 8 hours, the numbers of bacterial cells from all strains were normalized to approximately 5×10^8^ CFU ml^-1^ with fresh LB, followed by six 10-fold serial dilutions. Plate assays of cadmium tolerance with the *E. coli* cells expressing wild type RBP (from 10^-3^ to 10^-8^ dilution) were spotted 10 μL of each onto LB agar plates containing 50 µg mL^-1^ ampicillin and 0-180 μM cadmium concentrations. Then 10 μL of each strain of bacterial samples expressing RBP variants were spotted onto the medium containing 50 µg mL^-1^ ampicillin and 0 μM and 150 μM cadmiums. All plates were incubated at 37 °C for 16 hours before being read.

**Protein expression and purification**

The recombinant plasmids containing wild type RBP, cadmium binding proteins and their single alanine substitution mutant genes in pET-28a were transformed individually into BL21 (DE3) strains for proteins expression. The cells were grown overnight at 37 °C and one liter of LB was inoculated with 10 mL overnight culture, cultivating until the OD_600_ reached 0.6. The cultures were induced with 0.5 mM IPTG and incubated at 28 °C for 8 hours before harvesting. Cells were resuspended and lysed in lysis buffer containing 50 mM Tris-HCl (pH 8.0), 200 mM NaCl and 10 mM imidazole,1mM dithiothreitol (DTT), 1 mM phenylmethyl sulfonyl fluoride (PMSF). Cell lysates were centrifuged and the supernatants were loaded onto HisTrap (GE Healthcare Life Sciences). The N terminal 6*His-tag was removed by on column digestion using thrombin protease at 4 °C overnight. The target protein was eluted with 50 mM Tris-HCl, 200 mM NaCl, 10 mM imidazole as elution buffer followed by a Sephacryl S-200 HR column for further purification. The proteins with FRET pairs were expressed and purified with similar method.

**Isothermal titration calorimetry (ITC) assay**

The sample cell was loaded with 100 µM proteins dissolved in high purity 20 mM HEPES (pH 7.3), 150 mM NaCl and 1 mM TCEP each time, and 40 µL 1 mM CdCl_2_ or other metal ions in the same buffer was added automatically by syringe. The titration was performed with an initial 0.4 μL injection in 0.8 s, followed by nineteen 2 μL injections in 4 s with a reference power of 5 μcal/s at 25 °C. The spacing between each injection was 150 s, and the stirring speed during the titration was 1000 rpm. The control experiment was carried out for correction for ligand dilution and other nonspecific interactions by using the same concentration of corresponding metal ions and buffer was subtracted as reference in the original data.

**Microfluidic experiments**

The microfluidic chip used in this study was developed based on a microfluidic device developed previously(*2*). A schematic representation of the device is shown in Figure 4(a). It contains twenty-four separate microchannels. Each channel is divided into four parts: observation channel, agarose channel, source hole and sink hole. The observation channel is 600 μm in length, 200 μm in width and in 18 μm in height. The Agarose channel is 150 μm in length, 5 μm in height and 200 μm in width. When agarose plugs were constructed into the agarose channel, the convection current was avoided.

Different concentrations of metal ions were added into the source hole, which diffused into the channel to generate stable linear concentration gradients. In general, the chemical concentration in the microchannel can be described by the one dimensional diffusion equation(*3*). The concentration gradient is linear:

$\nabla C=C_{0}/l$ (Eq. S1),

where *C_0_* is the source hole concentration, l is the length of the microchannel. The linear concentration gradient is stable during the period of experiment. The stable time t for the linear concentration gradient can be estimated by the equation:

$\Delta C=t_{2}Dsdc/dl$ (Eq. S2),

where *V* is the volume of the source hole, s is the cross-sectional area between source hole and sink hole. The concentration gradients are very stable during the entire period of experiments. This process allowed us to observe responses of cells under different environmental conditions continuously.

The microfluidic devices were bonded to clean microscope cover classes (Fisher Scientific) after treating with oxygen plasma for 2 min in a plasma cleaner (Harrick Plasma) to create hydrophilic devices. Then 4 % agarose solution was loaded into each agarose channel at the room temperature. Minimal salt buffer (also termed blank buffer; 10 mM HEPES, 0.01 mM l-methionine, 10 mM sodium dl-lactate, pH 7.0) was loaded into the sink hole and the source holes to fill the chip.

Single colonies of microfluidic experiment used *E. coli* strains were incubated at 34 °C, 250 rpm overnight in Tryptone Broth medium (TB, 10g L^-1^ tryptone and 5 g L^-1^ NaCl) supplemented with 50 µg mL^-1^ ampicillin, 17 µg mL^-1^ chloramphenicol. The cultures were then diluted with 1:100 ratio by fresh TB medium containing antibiotics and 20 µM sodium salicylate and grew at 34 °C, 250 rpm until OD_600_ reached 0.3. Cells were harvested by centrifuge at 3000 rpm for 5 min. The pelleted cells were washed twice and resuspended in minimal salt buffer. All the metal ions used in the microfluidic experiments were dissolved and diluted in minimal salt buffer (pH 7.0). The prepared *E. coli* cells were added into the sink hole. After about 15 min, the cells diffused into the observation channel to reach a steady state. Images were captured to record the initial cell fluorescent intensities. After metal ion solutions were added into the source holes, the images of the observation channels were captured every 5 min for 90 min. The response of the cells was quantified by the fluorescent intensities of the analysis region using the Image J software (3). The relative fluorescent signal was calculated by comparison of the intensities at each time point with that of t = 0 min in the analysis region within the same Cd^2+^ concentration source hole.

**Analysis of protein expression in periplasmic region by osmotic shock procedure**

Cells were grown at 37 °C in 3 mL fresh LB medium and induced with 20 µM sodium salicylate. The cells were harvested by centrifuge (4500 rpm, 5 min) when the cell density reaching at OD_600_ = 1.0 and suspended in 1 mL 10 mM Tris-acetate (pH 7.3), 30 mM NaCl. Centrifuge (12000 rpm, 2 min) the cells and resuspend the pellet in 200 μL 30 mM Tris-acetate (pH 7.3). After that, add 200 μL 30 mM Tris-acetate (pH 7.3), containing 40 % sucrose following with 4 μL 10 mM EDTA and incubate 10min at room temperature. After centrifuge (12000 rpm, 2 min), resuspended the cells quickly using 600 μL ice-cold MgCl_2_ (0.5 mM) and incubate them in ice-water mixer for 10min before centrifuge. Remove and save the supernatant. Samples were then analyzed by 10 % SDS-polyacrylamide gel electrophoresis (PAGE).

**SI References**

1. J. Quan, J. Tian, Circular polymerase extension cloning for high-throughput cloning of complex and combinatorial DNA libraries. Nat Protoc 6, 242-251 (2011).

2. G. W. Si, W. Yang, S. Y. Bi, C. X. Luo, Q. Ouyang, A parallel diffusion-based microfluidic device for bacterial chemotaxis analysis. Lab Chip 12, 1389-1394 (2012).

3. Rasband, W.S., ImageJ, U.S. National Institutes of Health, Bethesda, Maryland, USA, https://imagej.nih.gov/ij/, 1997-2018.
